# Comparative Effectiveness of Peroral Endoscopic Myotomy (POEM) Versus Traditional Treatment Modalities for Achalasia: A Systematic Review

**DOI:** 10.7759/cureus.71917

**Published:** 2024-10-20

**Authors:** Malik Kasapoglu, Syeda Noor Us Saba, Ava Hashemi, Malaika Panchal, Safeera Khan

**Affiliations:** 1 Internal Medicine, California Institute of Behavioral Neurosciences and Psychology, Fairfield, USA; 2 Medicine, Bahçeşehir University, Istanbul, TUR; 3 Ophthalmology, California Institute of Behavioral Neurosciences and Psychology, Fairfield, USA; 4 College of Medicine, California Institute of Behavioral Neurosciences and Psychology, Fairfield, USA; 5 Medical Oncology, California Institute of Behavioral Neurosciences and Psychology, Fairfield, USA

**Keywords:** achalasia, esophageal myotomy, heller's myotomy, lower esophageal sphincter (les), peroral endoscopic myotomy (poem)

## Abstract

Achalasia is a rare esophageal motility disorder characterized by impaired relaxation of the lower esophageal sphincter (LES) and absence of peristalsis, leading to significant swallowing difficulties and other symptoms. Traditional treatment options, including Heller myotomy (HM) and pneumatic dilation (PD), have been effective but are associated with risks such as perforation and gastroesophageal reflux disease (GERD). Peroral endoscopic myotomy (POEM) has emerged as a minimally invasive alternative, potentially offering several advantages over conventional methods. This systematic review aims to compare the efficacy, safety, and long-term outcomes of POEM versus HM in the treatment of achalasia. We systematically reviewed studies that compared POEM with HM in achalasia patients, focusing on key outcomes such as myotomy length, operative time, treatment success rates, and complication rates. The review included 15 studies comprising four randomized controlled trials, 10 cohort studies, and one case-control study. The results consistently showed that POEM achieved longer myotomy lengths and shorter operative times compared to HM. POEM also demonstrated higher or comparable treatment success rates, with a uniform definition of success based on achieving an Eckardt score of ≤3. However, the complication rates, particularly the incidence of GERD, varied between the two procedures, highlighting the need for careful patient selection and long-term follow-up. POEM offers a promising alternative to HM for the treatment of achalasia, with advantages in terms of reduced invasiveness, shorter operative times, and potentially higher treatment success rates. However, further high-quality research is necessary to fully establish its long-term efficacy and safety compared to conventional treatments.

## Introduction and background

Achalasia is a rare gastrointestinal disorder characterized by aperistalsis and the failure of the lower esophageal sphincter (LES) to relax during swallowing [1,2]. While the exact etiology remains unclear, the condition is thought to result from the loss of inhibitory innervation of the myenteric plexus. Possible causes of this damage include autoimmune mechanisms and idiopathic factors. Additionally, various genetic predispositions, environmental influences [3], and viral infections have been implicated in the pathogenesis of achalasia [4]. This disorder affects individuals of all ages and both sexes, with an incidence of approximately 2.92 per 100,000 adults and 0.11 per 100,000 children, and a male-to-female ratio of 1:1 [5]. Despite its rarity, achalasia is one of the most common motor disorders causing dysphagia [6].

Diagnosis of achalasia typically involves a combination of clinical evaluation and diagnostic imaging techniques, such as esophagram or barium swallow, along with esophageal manometry, which measures the function and pressure of the esophageal muscles [7]. The primary goal of treatment is to relieve the obstruction at the LES, thereby alleviating dysphagia and preventing food regurgitation [8]. Medical treatment options include botulinum toxin injections, calcium channel blockers, and other medications aimed at inhibiting the cholinergic innervation of LES muscles [9]. Historically, surgical myotomy and pneumatic dilation (PD) have been the cornerstone treatments for improving esophageal emptying and addressing dysphagia in patients with achalasia. Pneumatic dilation, a highly effective endoscopic procedure, involves the use of an air-filled balloon to forcibly stretch and disrupt the LES fibers [10]. This procedure is favored for its simplicity, reproducibility, minimal complications, and low mortality rate​ [11]. Heller’s myotomy, another well-established surgical option, involves cutting the LES muscles to relieve the obstruction, aiming for long-term symptom control and improved esophageal function [12]. However, the risk of complications such as perforation and gastroesophageal reflux disease (GERD) has led to the exploration of alternative treatments.

Peroral endoscopic myotomy (POEM) represents a relatively new advancement in the treatment of achalasia [13]. Utilizing a submucosal tunneling technique, POEM allows precise myotomy of the LES without the need for external incisions [14]. Compared to traditional surgical methods, POEM offers several potential advantages, including reduced invasiveness, shorter hospital stays, and possibly faster recovery times [15]. However, further research is needed to comprehensively evaluate how POEM compares to conventional treatments in terms of symptom relief, esophageal function improvement, and procedural safety.

## Review

Rationale

Achalasia is a complex and debilitating esophageal motility disorder that presents significant challenges in clinical management. Traditional treatments like Heller myotomy (HM) and pneumatic dilation (PD) have been the mainstays of therapy, providing effective symptom relief for many patients. However, these treatments are associated with notable risks, including the potential for perforation and the development of gastroesophageal reflux disease (GERD). The advent of peroral endoscopic myotomy (POEM) offers a novel approach, potentially overcoming some of the limitations of traditional methods by providing a minimally invasive alternative with the promise of shorter recovery times and reduced complications. Despite the growing adoption of POEM, there remains a need for comprehensive evidence comparing its efficacy, safety, and long-term outcomes with those of established treatments like HM.

Objectives

The primary objective of this systematic review is to critically evaluate and compare the efficacy and safety of POEM with Heller myotomy in the treatment of achalasia. Specific objectives include:

1) To assess the differences in myotomy length between POEM and HM and their impact on treatment outcomes. 2) To compare the median operative times between POEM and HM, evaluating the efficiency and potential benefits in terms of reduced procedural time. 3) To compare the success rates of POEM and HM in achieving symptom relief and improving esophageal function, with a focus on long-term outcomes. 4) To analyze the incidence of complications, including perforation and GERD, associated with POEM versus HM.

Methods

Study Design

This research followed the recommendations made by the Preferred Reporting Items for Systematic Review and Meta-Analyses (PRISMA) guidelines [16]. The Population, Intervention, Comparison, Outcome, and Study Design (PICOS) scheme was followed. All of these parameters are highlighted in the inclusion and exclusion criteria listed below (Table 1).

**Table 1:** Eligibility criteria for the systematic review RCT: Randomized Controlled Trial; POEM: Peroral Endoscopic Myotomy

Criteria	Inclusion Criteria	Exclusion Criteria
Language	English	All other languages
Timeframe of publications	2020-2024	Older
Studies Included	Retrospective Cohort studies, case-control studies, RCTs	Perspective Reviews Gray literature
Region	All	
Target Population	Patients diagnosed with esophageal achalasia.	Patients with achalasia-like symptoms but without a confirmed diagnosis.
Context	Evaluation of the comparative Effectiveness of Peroral Endoscopic Myotomy (POEM) Versus Traditional Treatment Modalities for Achalasia	

Eligibility Criteria

The inclusion criteria were as follows: (1) peer-reviewed studies that compared the efficacy of POEM to other traditional modalities; (2) studies that were published between 2018 and 2024; (3) studies with available abstracts and full-free texts; (4) studies with comparative designs, either randomized control trials (RCTs) or observational comparative studies (cohort, case-control); (5) studies published in English; (6) studies reporting outcomes related to treatment efficacy (i.e., success, dysphagia improvement) or safety (i.e., complications) of the surgical therapies.

The exclusion criteria were as follows: (1) all studies published before 2018; (2) narrative reviews, Grey literature, and poor study designs (i.e., case series, case reports); (3) people with achalasia-like symptoms but without a confirmed diagnosis; (4) studies reported in languages other than English; (5) studies reporting insufficient data on the outcomes related to the research objectives.

Search Strategy

Several digital databases were searched for the relevant literature on the comparative effectiveness of POEM with other traditional modalities. The search was done on May 5th, 2024, through PubMed and Google Scholar [17,18]. We also searched independent journals and other sources of literature [44]. In addition to these, digital databases were also considered. For the search strategy, the boolean operators (AND/OR) were used. We wanted the search for the articles to be more specific; therefore, we applied multiple filters to the search engines as follows: ("peroral endoscopic myotomy" OR "POEM") AND ("traditional treatment" OR "conventional treatment" OR "standard treatment") AND "achalasia" AND ("comparative effectiveness" OR "comparative study" OR "comparison"). Following the established inclusion criteria, a team of researchers searched peer-reviewed journals and publications from the relevant literature. To lessen the likelihood of publication bias, we looked further into peer-reviewed journals with a high impact factor. Additionally, the reference lists of studies that were finally selected for this systematic review were also examined. A list of articles was gathered in case they were chosen for this review.

Study Selection

Retrieved studies from searched databases were imported into EndNote software for duplicate identification. Following duplicate removal, the remaining records were uploaded into Rayyan Software for formal screening [19]. The selection of articles was based on our pre-defined eligibility criteria following a three-phase screening approach (title, abstract, and full text). 

Data Extraction

Following the identification of studies to be finally included in this review, the senior author constructed an extraction sheet using Microsoft Excel software. The sheet contained the data in two tabs. The first part covered the baseline characteristics of included studies (authors' names, year of publication, journal name, study design, country of investigation, and follow-up period). The second part covered the main outcomes addressed in the included studies by comparing various treatment modality efficacy (dysphagia improvement, success rate, etc.) and safety (complications, etc.) endpoints. Extracted data were validated by the senior author before data synthesis.

Risk of Bias (RoB) and Methodological Quality Assessment

The methodological quality of the included studies was evaluated using established assessment tools. For retrospective observational studies, the Newcastle-Ottawa Scale (NOS) was used, which assesses the selection of study groups, comparability of cohorts, and ascertainment of outcomes. For RCTs, the Cochrane Risk of Bias tool was applied, evaluating domains such as randomization, allocation concealment, blinding, and completeness of outcome data. The quality assessments helped to identify potential biases and determine the overall reliability of the synthesized data.

Data Synthesis

A narrative synthesis was conducted to qualitatively summarize the findings of the included studies. This involved comparing and contrasting the various treatment modalities, discussing the methodologies and contexts of the studies, and highlighting the main themes and patterns observed in the data. The qualitative synthesis provided a detailed overview of the characteristics and outcomes of POEM versus HM in treating esophageal achalasia.

Results

Database Search and Screening Results

**Figure 1:**
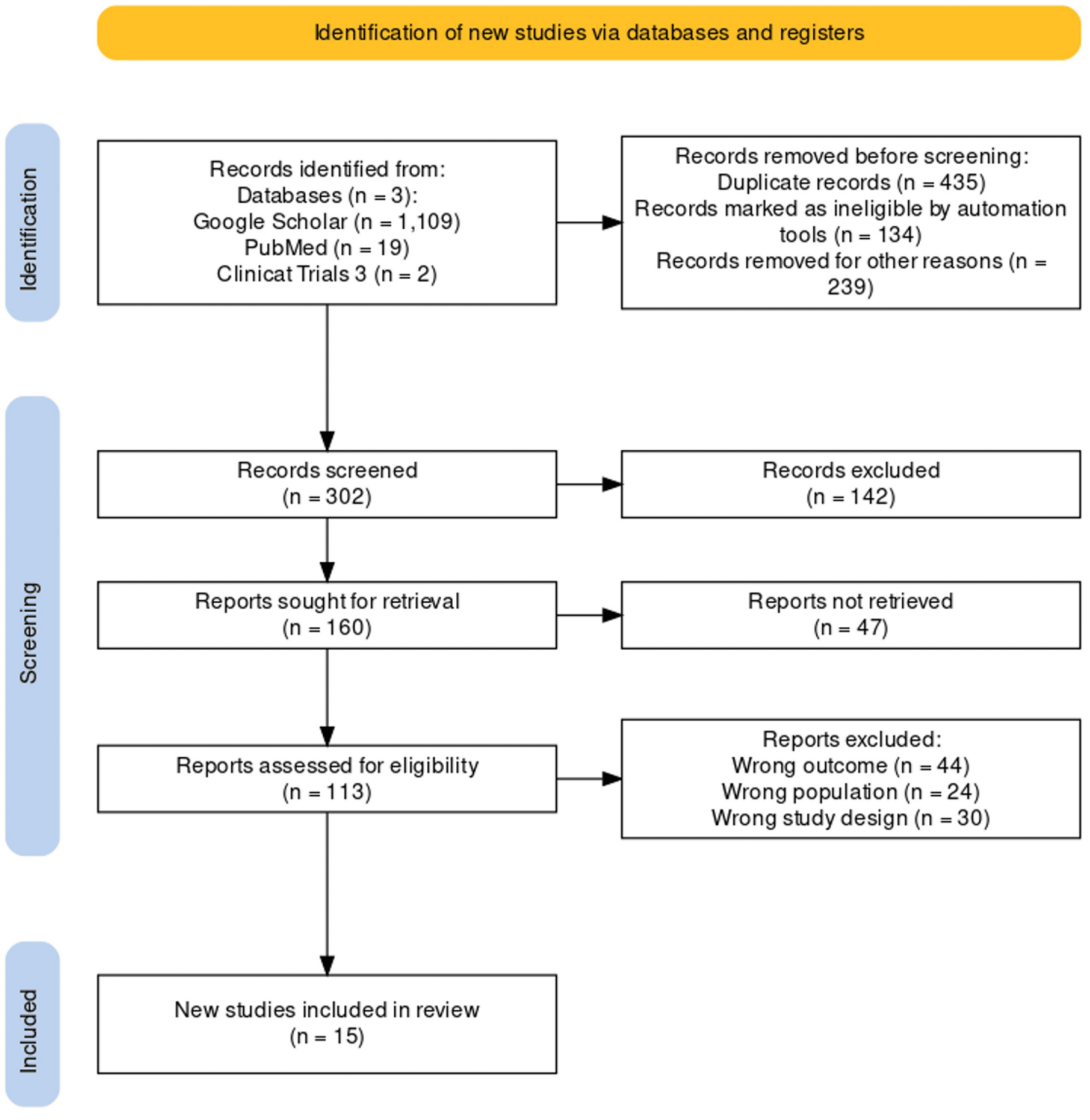
PRISMA flow chart for selected items n: number of records; PRISMA: Preferred Reporting Items for Systematic Reviews and Meta-Analyses

The results of the database search and screening processes are illustrated in the PRISMA flow diagram as recommended by the PRISMA guidelines (Figure 1) [16].

In summary, 1130 articles were retrieved from searched databases, of which 828 articles were excluded. A total of 302 articles underwent formal screening, of which 160 articles underwent full-text screening. Unfortunately, the full text of 47 articles could not be retrieved. Attempts to contact the first and corresponding authors of these articles were made through emails and ResearchGate; however, no response was received. Eventually, 113 articles were screened, of which 98 studies were excluded for the following reasons: wrong outcome (n = 44), wrong population (n = 24), and wrong study design (n = 30). Finally, 15 articles were deemed eligible for data synthesis in this review.

Characteristics and Reported Outcomes of Included Studies

Table 2 of this systematic review compares POEM and HM based on three different variables. These variables are median operative timings, myotomy length, and overall treatment success rate.

**Table 2:** Patient characteristics and study endpoints POEM: per-oral endoscopic myotomy; HM: Heller’s myotomy; NR: not reported

Study ID.	Participants			Median myotomy length		Scale of Variable Assessment	Treatment success rate		Median operative time	
	Total	POEM	HM	POEM	HM		POEM	HM	POEM	HM
Shally et al. (2022) [20]	58 patients	33	25	11cm	8cm	Eckardt ≤ 3	88%	76%	106 min	145 min
Wong et al. (2022) [21]	60 patients	63	60	10.1 cm	6.2 cm	NR	NR	NR	125.9	144.1
Mussies et al. (2023) [22]	12 patients	12	9	NR	NR	Eckardt score > 3	100%	66%	NR	NR
De Moura et al. (2022)[23]	40 patients	20	20	NR	NR	dysphagia score ≥ II and Eckardt score > 3	95%	100%	95.70 ± 30.47 min	296.75 ± 56.13
Sarıcı et al. (2023) [24]	52 patients	17	35	11 cm	5 cm	Eckardt score ≤ 3 at 1 year	82.40%	85.70%	NR	NR
Huang Z et al. (2022) [25]	75 patients	37	38	8cm	8cm	Eckardt score ≤ 3 at 12-month follow-up	94.60%	92.10%	40.6 ± 8.4	42.5 ± 8.5
Podboy et al. (2020) [26]	98 patients	55	43	10.1+/−4.2	6.9+/−1.5	Eckardt Score > 3 for at least 4 weeks	72.70%	65.10%	NR	NR
Saleh et al. (2023) [27]	90 patients	45	45	NR	NR	Eckardt score >3 and substantial stasis (≥2 cm)	62.2	26.7	NR	NR
Zhang Z et al. (2023) [28]	219 participants	88	131	9.8 ± 2.0 cm	NR	Eckardt scores	90.70%	NR	NR	NR
Sudarshan et al. 2022 [29]	518 patients	210	308	7cm	NR	Eckardt score	NR	NR	85 minutes	NR
Costantini A et al. 2020 [30]	140 patients	70	70	NR	NR	Eckardt scores	99.30%	97.70%	NR	NR
Shea et al. (2019) [31]	141 patients	44	97	NR	NR	Eckardt dysphagia scores.	73.30%	65.40%	NR	NR
Gu et al. (2019) [32]	94 patients	48	46	NR	NR	Eckardt scores	93.80%	95.70%	45.6 ± 16.2 minutes	31.2 ± 15.3
Mangiola et al. (2018)[33]	26 patients	26 children	NR	10 (2.6)	NR	Eckardt score 7.2	33.2 mmHg	NR	56.2 minutes	NR
Ward et al. (2020) [34]	100 patients	54	56	NR	NR	DeMeester scores	24.6 to 11.5mmHg	22.8 to 11mmHg	NR	NR

Meanwhile, Table 3 indicates the study ID, the location where the research was conducted, the study design, and the follow-up period along with the results of each study mentioned. After the study section was finished, the study interventions were tabulated against their respective study populations and results individually. Only the themes that are relevant to the results are mentioned in the synthesis table.

**Table 3:** Results of the systematic review USA: United States of America; HM: Heller's myotomy; POEM: per-oral endoscopic myotomy; EBD: endoscopic balloon dilation; P: p-value; pneumatic dilation; ITT: intention-to-treat

Study ID.	Location	Study Design	Follow up time	Results
Shally et al. (2022) [20]	USA	Retrospective Cohort study	2014 and 2021	POEM has shorter operative median timings, longer myotomy length, and a better treatment success rate overall than HM.
Wong et al. (2022) [21]	Malaysia	Retrospective Cohort study	January 2010 to April 2021	The complication rate of POEM was very low as compared to HM.
Mussies et al. (2023) [22]	Netherlands	Randomized Control Trial	26–40 months	POEM has been shown feasible and safe in children [11, 12, 17]. In a randomized controlled trial in adults comparing the efficacy of POEM vs EBD, no serious adverse events occurred after POEM
De Moura et al. (2022) [23]	Brazil	Randomized Control Trial	March 2017 and February 2018	At 12 months follow-up, no statistically significant differences were found regarding symptom management (P = 0.192, P = 0.242, and P = 0.242, respectively).
Sarıcı et al. (2023) [24]	USA	Retrospective Cohort study	2013 and 2021	To alleviate EGJOO symptoms, peroral endoscopic myotomy and Heller myotomy combined with Dor fundoplication perform equally well.
Huang Z et al. (2022) [25]	China	Randomized Control Trial	May 2017 to December 2018	The rates of clinical success in the ITT population were 92.1% in the conventional group and 94.6% in the snare group at the 12-month follow-up
Podboy et al. (2020) [26]	USA	Retrospective Cohort study	follow-up of 3.94 years,	Overall long-term success did not differ statistically (POEM success rate was 72.7%, while HM had a 65.1%,
Saleh et al. (2023) [27]	Netherlands	Randomized Control Trial	January 2014 to June 2020	When the primary outcome was analyzed, patients treated with POEM (28 of 45 patients, or 62.2%) had better treatment success at the 1-year follow-up than patients treated with PD (12 of 45 patients, or 26.7%).
Zhang Z et al. (2023)[28]	China	Retrospective Cohort study	August 2011 and August 2021	POEM had better treatment success rates than other conventional modalities because it provides long-term symptom relief.
Sudarshan et al. 2022 [29]	USA	Retrospective Cohort study	April 2014 and July 2019.	POEM ought to be taken as first-line therapy in this difficult disease because of its low morbidity profile.
Costantini A et al. 2020 [30]	Italy	Case-control study	January 2014 to November 2017	At 4 years follow-up, the probability of having symptoms adequately controlled was > 90% for both groups after the treatment.
Shea et al. (2019)[31]	USA	Retrospective Cohort study	2009 to 2018	Both POEM and HM had mean Eckardt score <3 which demonstrates a successful myotomy through both treatment modalities.
Gu et al. (2019) [32]	China	Randomized Control Trial	February 2018 and February 2019	The standard myotomy group experienced a higher frequency of postoperative aberrant esophageal acid exposure compared to the short myotomy group (21/48 patients [43.8%] vs 11/46 patients [23.9%], P =.042).
Mangiola et al. (2018) [33]	Italy	Retrospective Cohort study	January 2012 to June 2017	POEM is a minimally invasive therapy used to treat children patients with achalasia. Our findings support POEM's long-term effectiveness in children when there are no serious side effects.
Ward et al. (2020) [34]	USA	Retrospective Cohort study	January 2015 to December 2019	POEM patients had a lower incidence of postoperative complications as compared to HM.

One method of reducing bias in the analysis was to (1) select the best research by conducting a literature review, (2) mandate the disclosure of conflicts of interest in peer reviews, (3) get rid of biases in clinical research and practice about informed consent and peer review, (4) replace the ordinary review articles, and (5) routinely remove systematic reviews and narrative reviews from the literature to uphold the study's standards. These recommendations identify and remove study protocol bias following the steps for reducing publication bias.

Quality Assessment of Cohort Studies

We have utilized the NOS scale to evaluate the quality of the retrospective observational studies that were included in the synthesis table. All of the 10 included papers were retrospective cohort studies. According to Hartling et al. (2013) [35], the NOS provided a systematic framework for assessing the methodological quality of each study. The NOS was chosen because of its ability to comprehensively evaluate critical elements of research quality, including study group selection, group comparability, and outcome determination. By assessing these categories, the NOS enabled a qualitative understanding of the advantages and disadvantages of each study, hence facilitating well-informed decision-making in the synthesis and interpretation of study results. The results of the quality assessment are provided in the table below (Table 4).

**Table 4:** The methodological quality of included retrospective cohort studies using the Newcastle Ottawa Scale (NOS) NA: not applicable. The number of stars corresponds to the methodological quality in each domain. Good quality: 3 or 4 stars in selection domain AND 1 or 2 stars in comparability domain AND 2 or 3 stars in outcome/exposure domain; Fair quality: 2 stars in selection domain AND 1 or 2 stars in comparability domain AND 2 or 3 stars in outcome/exposure domain; Poor quality: 0 or 1 star in selection domain OR 0 stars in comparability domain OR 0 or 1 star in outcome/exposure domain

Study Id.	Selection of cohorts				Comparability of cohorts	Outcome			Total Score
	Representativeness of the exposed cohort	Selection of the non-exposed cohort	Ascertainment of exposure	Demonstration that outcome of interest was not present at the start of the study	Comparability of cohorts based on the design or analysis	Assessment of outcome	Was follow-up long enough for outcomes to occur	Adequacy of follow-up of cohorts	
Shally et al. (2022) [20]	☆	☆	☆	☆	☆	☆	☆	☆	8/10
Wong et al. (2022)[21]	☆	☆	NA	☆☆	☆	☆☆☆	NA	☆	9/10
De Moura et al. (2022) [23]	☆	☆	☆	☆	☆	☆	☆	☆	8/10
Sarıcı et al. (2023) [24]	☆	☆	☆	☆	☆	NA	☆	☆	7/10
Podboy et al. (2020) [26]	☆	NA	☆	☆	☆☆	☆	☆	☆	8/10
Zhang Z et al. (2023) [28]	☆	☆	NA	☆	☆	☆	NA	☆	6/10
Sudarshan et al. (2022) [29]	☆	☆	☆	☆☆	☆	☆	☆	☆	9/10
Costantini A et al. (2020) [30]	☆	☆	☆	NA	☆	☆	☆	☆	7/10
Mangiola et al. (2018) [33]	☆	☆	☆	☆	☆	NA	☆	☆	7/10
Ward et al. (2020) [34]	☆☆	☆	☆	☆	☆	☆	☆	☆	9/10

Risk of Bias Assessment of RCTs

**Figure 2:**
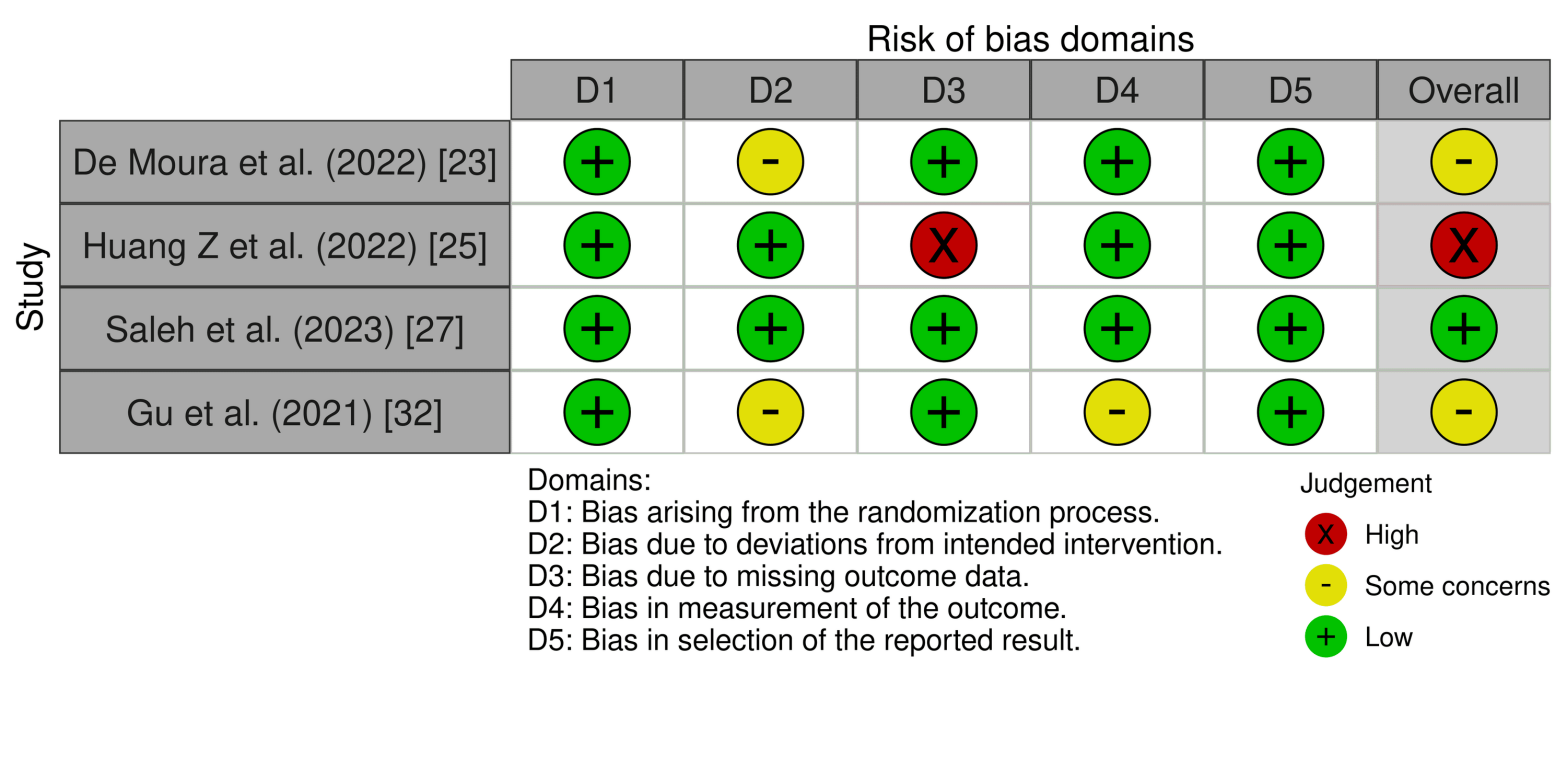
A summary of the risk of bias of included randomized controlled trials based on the revised Cochrane tool

The Cochrane criteria for ROB were used to independently identify all of the papers that qualified for the analysis. We calculated the ROB via the Cochrane Risk-of-Bias (version 2019) online tool [22]. According to the Cochrane protocol, the risk of bias algorithm assessed five domains of potential risk of bias. These domains were as follows: (i) bias due to the randomization process; (ii) deviation from intended intervention; (iii) missing outcome data; (iv) measurement of the outcome; (v) selection of the reported result. Overall, two RCTs had some concerns, while one trial had a low risk of bias and the remaining trial had a high risk of bias (Figure 2). 

A Descriptive Summary of Included Studies

A total of 15 studies [20-34] were included in this systematic review, comprising four RCTs, 10 cohort studies, and one case-control study. Notably, all cohort studies utilized a retrospective design. The sample sizes across the studies ranged from as few as 26 to as many as 518 patients diagnosed with achalasia. The following results section presents a comparative analysis of POEM versus HM across various outcome measures, including myotomy length, median operative time, and treatment success rates.

Myotomy Length

The length of the myotomy is a critical determinant of the therapeutic success of myotomy procedures. In the reviewed studies, the myotomy length for POEM was generally longer than that for HM, which potentially contributed to better disruption of the lower esophageal sphincter (LES) muscle and more effective symptom relief.

For instance, Shally et al. (2022) reported a median myotomy length of 11 cm for POEM compared to eight cm for HM [20]. Similarly, Wong et al. (2022) found a median myotomy length of 10.1 cm for POEM, while HM was 6.2 cm [21]. Sarıcı et al. (2023) and Zhang et al. (2023) also confirmed longer myotomy lengths for POEM, with measurements of 11 cm and 9.8 cm, respectively [24,28], compared to shorter lengths achieved in HM. These findings were consistent across other retrospective cohorts and RCTs, suggesting that the longer myotomy length associated with POEM may lead to improved esophageal function and higher success rates compared to HM.

Median Operative Time

Median operative time is a crucial factor in assessing procedural efficiency and resource utilization. In the included studies, POEM was generally associated with shorter operative times compared to HM, which may translate to faster recovery and reduced healthcare costs.

For example, Shally et al. (2022) reported a median operative time of 106 minutes for POEM, significantly shorter than the 145 minutes for HM [20]. Similarly, De Moura et al. (2022) reported a median operative time of 95.7 minutes for POEM, compared to 296.75 minutes for HM [23]. RCTs, such as those conducted by Huang et al. (2022) and Gu et al. (2019), also demonstrated shorter operative times for POEM, with respective times of 40.6 and 45.6 minutes compared to 42.5 and 31.2 minutes for HM, respectively [25,32]. These findings indicate that POEM may offer advantages in terms of operative efficiency over HM.

Treatment Success Rate

Treatment success rates, often measured by symptom resolution and improvement in functional outcomes, are a key indicator of the effectiveness of POEM and HM. The studies included in this review consistently showed that POEM was associated with higher or comparable success rates compared to HM.

For instance, Mussies et al. (2023) reported a treatment success rate of 100% for HM, slightly higher than the 95% for POEM [22]. However, most other studies reported higher success rates for POEM. Shea et al. (2019) demonstrated a success rate of 73.4% for POEM, compared to 65.4% for HM [31]. Shally et al. (2022) similarly reported an 88% success rate for POEM versus 76% for HM [20]. Moreover, Zhang et al. (2023) highlighted that POEM provided long-term symptom relief and had better treatment success rates than other conventional modalities [28].

Overall, the included studies suggest that POEM is generally associated with longer myotomy lengths, shorter operative times, and higher treatment success rates compared to HM, making it a potentially superior option for the treatment of achalasia.

Discussion

Although there are several meta-analyses that discuss the efficacy of POEM in the treatment of achalasia [36,37], the results have been inconsistent and contradictory [38-40]. This systematic review provides a comprehensive evaluation of POEM compared to HM in treating achalasia. The findings across the included studies indicate that POEM may offer several advantages over HM in terms of myotomy length, operative time, and treatment success rates. These findings align with the evolving understanding of achalasia management, where POEM is increasingly being recognized as a viable alternative to the more traditional HM.

Myotomy Length and Its Implications

The length of the myotomy is a crucial factor in the success of achalasia treatment. Longer myotomies, as typically achieved with POEM, are associated with more effective disruption of the LES, leading to better symptom relief. The reviewed studies consistently demonstrated that POEM resulted in significantly longer myotomies compared to HM. For instance, Shally et al. (2022) and Sarıcı et al. (2023) reported median myotomy lengths of 11 cm for POEM, whereas the myotomy lengths for HM were consistently shorter, ranging from five to eight cm, respectively [20,24].

This consistent observation of longer myotomy lengths with POEM suggests that this technique may provide a more thorough alleviation of the LES dysfunction characteristic of achalasia. The potential for better symptom resolution with longer myotomies was further supported by studies like Zhang et al. (2023), which demonstrated that longer myotomies are correlated with improved esophageal function and reduced symptom recurrence [28]. However, it is important to consider the balance between achieving sufficient myotomy length and avoiding unnecessary extension, which may increase the risk of complications such as GERD [41]. Future studies should continue to explore the optimal myotomy length that maximizes therapeutic benefit while minimizing adverse effects.

Operative Time and Efficiency

Operative time is a significant consideration in surgical and endoscopic procedures, as shorter procedures are generally associated with faster recovery times and reduced healthcare costs [42]. The studies included in this review consistently showed that POEM had a shorter operative time compared to HM. For example, De Moura et al. (2022) reported that the median operative time for POEM was 95.7 minutes, significantly shorter than the 296.75 minutes required for HM [23].

This difference in operative time may be attributed to the minimally invasive nature of POEM, which allows for a quicker and more streamlined procedure. Additionally, the shorter operative time associated with POEM may contribute to a lower overall complication rate, as shorter procedures reduce the duration of anesthesia and the likelihood of intraoperative complications. Nevertheless, the variability in operative times across different studies highlights the need for standardized procedural protocols and further research to identify factors that may influence operative time, such as surgeon experience and patient characteristics.

Treatment Success Rates and Long-Term Outcomes

Treatment success rates are a critical measure of the efficacy of POEM and HM, reflecting the ability of these procedures to achieve symptom resolution and improve patient quality of life [43]. One of the strengths of the studies included in this review is the consistent definition of treatment success across all studies, which was based on achieving an Eckardt score of ≤3. This uniformity in defining success allows for more accurate comparisons across different studies and strengthens the validity of the findings.

The studies reviewed here suggest that POEM generally achieves higher or comparable treatment success rates compared to HM. For instance, Shally et al. (2022) reported a success rate of 88% for POEM, which was higher than the 76% success rate observed for HM [20]. Similarly, Shea et al. (2019) demonstrated a treatment success rate of 73.4% for POEM compared to 65.4% for HM [31].

While these findings support the efficacy of POEM, it is essential to note that the success rates across studies varied, with some studies showing no significant difference between POEM and HM. For example, Mussies et al. (2023) reported a 100% success rate for HM compared to 95% for POEM, suggesting that HM remains a highly effective treatment option in certain patient populations [22]. The variation in success rates may be due to differences in study design, patient selection, and follow-up duration.

Another important consideration is the long-term sustainability of treatment success. Studies such as Zhang et al. (2023) emphasized the importance of long-term follow-up in assessing the durability of treatment outcomes [28]. While POEM offers the advantage of being less invasive and having a shorter recovery time, its long-term efficacy compared to HM, particularly in terms of preventing symptom recurrence and complications such as GERD, warrants further investigation. Future research should focus on long-term comparative studies with extended follow-up periods to better understand the durability of POEM outcomes.

Limitations and Future Directions

This systematic review has several limitations that should be acknowledged. First, the majority of included studies were retrospective cohort studies, which may introduce bias due to the reliance on historical data and lack of randomization. Additionally, the heterogeneity in study designs, patient populations, and outcome measures makes direct comparisons challenging. The relatively small sample sizes in some studies also limit the generalizability of the findings.

Despite these limitations, the evidence presented in this review supports the growing consensus that POEM is a promising alternative to HM for the treatment of achalasia. However, further high-quality RCTs with larger sample sizes and longer follow-up periods are needed to confirm these findings and to establish standardized protocols for POEM. Additionally, future studies should explore the impact of surgeon experience, patient selection, and adjunctive therapies on the outcomes of POEM to optimize its use in clinical practice.

## Conclusions

This systematic review provides a comprehensive comparison of peroral endoscopic myotomy (POEM) and Heller myotomy (HM) in the treatment of achalasia. The findings indicate that POEM offers several potential advantages over HM, including longer myotomy lengths, shorter operative times, and higher or comparable treatment success rates. These benefits, coupled with the minimally invasive nature of POEM, suggest that it may be a favorable alternative to traditional surgical methods for many patients. However, the variability in complication rates, particularly the risk of gastroesophageal reflux disease (GERD), underscores the importance of careful patient selection and individualized treatment planning. Despite the promising results, the need for further high-quality randomized controlled trials with larger sample sizes and long-term follow-up studies remains critical to fully establish the efficacy, safety, and durability of POEM compared to conventional approaches. As the field of achalasia treatment continues to evolve, POEM represents a significant advancement with the potential to improve patient outcomes, but it should be considered within the broader context of each patient's clinical profile and treatment goals.
